# Multi-User Low Intrusive Occupancy Detection

**DOI:** 10.3390/s18030796

**Published:** 2018-03-06

**Authors:** Azkario Rizky Pratama, Widyawan Widyawan, Alexander Lazovik, Marco Aiello

**Affiliations:** 1 Distributed Systems Group, Johann Bernoulli Institute for Mathematics and Computer Science, University of Groningen, Groningen 9747 AG, The Netherlands; a.lazovik@rug.nl (A.L.); m.aiello@rug.nl (M.A.); 2 Department of Electrical Engineering and Information Technology, Universitas Gadjah Mada, Daerah Istimewa Yogyakarta 55281, Indonesia; widyawan@ugm.ac.id

**Keywords:** occupancy detection, low-intrusive, Bluetooth Low Energy, BLE beacons, smart meter, sensor fusion

## Abstract

Smart spaces are those that are aware of their state and can act accordingly. Among the central elements of such a state is the presence of humans and their number. For a smart office building, such information can be used for saving energy and safety purposes. While acquiring presence information is crucial, using sensing techniques that are highly intrusive, such as cameras, is often not acceptable for the building occupants. In this paper, we illustrate a proposal for occupancy detection which is low intrusive; it is based on equipment typically available in modern offices such as room-level power-metering and an app running on workers’ mobile phones. For power metering, we collect the aggregated power consumption and disaggregate the load of each device. For the mobile phone, we use the Received Signal Strength (RSS) of BLE (Bluetooth Low Energy) nodes deployed around workspaces to localize the phone in a room. We test the system in our offices. The experiments show that sensor fusion of the two sensing modalities gives 87–90% accuracy, demonstrating the effectiveness of the proposed approach.

## 1. Introduction

In 2012, commercial and residential buildings accounted for 40% of the total energy consumption and were responsible for 36% of the EU total CO${}_{2}$ emissions [1]. Buildings’ emissions are higher than other sectors such as industrial and transportation and are projected to increase due to societal changes that entail more office related jobs [2]. In particular, commercial office buildings have the highest energy use intensity [3].

The application of occupant-driven energy control has a central role in improving the energy efficiency of some typical power consumptions in commercial office buildings, such as lighting, and heating, ventilation and air conditioning (HVAC). Intuitively, lighting and HVAC consumptions can be reduced for unoccupied spaces or adjusted based on the number of occupants. Nevertheless, such an effort is hampered due to the insufficient fine-grained occupancy information [4]. That is, a typical motion sensor (e.g., Passive Infrared or PIR sensor) does not support the counting and identification of peoples’ presence in a shared workspace (i.e., only detection of binary occupancy). Furthermore, the sensor generates many false positives which require the combination with other modalities [5]. In addition, these type of sensors are not sufficiently sensitive for low amplitude motions [6]. To understand the building occupancy context better, we need more detailed occupancy information, preferably using low-intrusive approaches.

We resort to occupancy extraction using device utilization information from electricity consumption. Power meters seamlessly measure the total power consumption of devices being used by users. They can be installed either one meter per appliance or one per circuit breaker per workspace. The energy monitoring per appliance is costly and has the higher level of intrusiveness than per room monitoring, as we need to install more power meters per users’ workspace. To this end, we use few sensors followed by breaking down the aggregated power. This approach is similar to Weiss et al. [7], though they do not disclose users’ presence. We take the approach one step further, by using room level consumptions to infer the presence of people in office spaces. In this way, we aim to achieve fine-grained user presence information.

By monitoring power consumption of, for instance, computer screens, we derive occupancy indications for a particular user in a shared workspace. We refer to this modality as *indirect sensing*, that is, an input that does not directly show the occupancy information but is an indication of a more complex process that can lead to conclusions about occupancy. The focus on computer screens is based on the observation that most of the time people in offices are engaged in computer-related activities. In the US, workers spend on average more than six hours per day at the computer [8].

As opposed to indirect sensing, *direct sensing* is an observation of a phenomenon that is explicitly affected by occupancy changes. In the present work, we consider both sensing modalities, and in particular, we resort to Bluetooth beaconing to observe user occupancy. The publication of the Bluetooth Low Energy (BLE) protocol and the many related, recent implementations point to a method to do localization based on a widespread technology such as Bluetooth. This is particularly interesting because BLE does not require the pairing of devices. To the extent that a Bluetooth module is activated and the beacon discovery service is on, one can receive small beacon packets from nearby emitters. BLE beacons deployment around workspaces can help to reveal the position relative to beacon references of personal user devices and, in turn, of the user. This process is known as localization based on BLE, a process of users’ location determination using received Bluetooth signal indication. However, there are external factors that affect signal-based positioning systems by worsening the performance. These include fast fading signals during propagation [9] and device heterogeneity that provides different readings for the same signal strength [10]. In the rest of this paper, we refer to direct and indirect sensing as BLE-beacon and room-level power meter (PM), respectively. The developed occupancy inference system will be referred to as dev-recog (short for device recognition) and BLE inference, respectively.

To the best of our knowledge, extracting an aggregated electricity consumption to reveal fine-grained contextual information (i.e., individual occupancy) is new. That is, several works have investigated occupancy status at a coarser granularity [11] or using a high number of power meters [6]. From the BLE perspective, several authors have used it for localization, e.g., [12,13]. Related works appear to have good portability, though we have a different aim, that is a richer contextual description such as multi-occupancy detection. In addition, our calibration step is lighter, in fact, we do not expect every user to give fingerprinting calibration. Instead, we only collect signal strength reference using one mobile phone.

The contribution of the present paper can be summarized as follows:

- A proposal of a novel personalized low intrusive occupancy detection approach in a shared workspace using room-level power meters and mobile phones;
- A model validation with a comparison to existing techniques;
- An approach fusing sensors data to improve accuracy, that is, simultaneously utilizing aggregated power consumption and BLE Received Signal Strength (RSS); and
- A validation of the approach with related feasibility assessment.

The reminder of the paper is organized as follows. We illustrate the design of the occupancy detection system and its implementation in Section 2. The experimental setup, metrics, and preliminary validation are described in Section 3. Results and Discussion are reported in Section 4 and Section 5, respectively. Finally, we present related work in Section 6 and concluding remarks in Section 7.

## 2. Design and Implementation

Occupancy detection in an office room can be seen as a classification problem. That is, given a set of various sensor input, decide whether the room is occupied and by how many people. In our work, there are two types of sensory modalities, namely, room-level PM and BLE-beacon. The room-level PM measures the power consumption of a shared workspace. Given an aggregated power load, occupancy can be indicated through the recognition of activated devices. Another sensory modality, BLE-beacon, shows occupancy through the location prediction based on the signal strength received by users’ mobile phones. These two sensory modalities are chosen as both are low-intrusive. That is, the user is in control of the application and there is no recording of videos and sound. A mobile phone only captures signals locally and does not share the data with a central server. In general, it does not have any knowledge about signals discovered by other mobile phones: we cannot infer multi-person occupancy using single BLE beacon measurement. Hence, we solve the multi-person occupancy problem as several individual one-person occupancy problems. The detail of sensor implementation and how it is translated into the occupancy output is described in the following.

### 2.1. Occupancy Inference from Power Consumption

Based on the fact that electric devices used by workers leave electricity fingerprints and that the device energy consumption pattern over time is known, we design a system for detecting workers’ occupancy from power consumption. To relate such consumption to an individual occupancy state, we make use of the inventory list of worker’s devices. This list defines what devices are owned by whom. Creating such list is a simple, one-off task as devices in a workspace are rarely changing. Given such knowledge, the task of occupancy inference shifts to the recognition of particular devices and their states.

There are several possible measuring points for looking at power consumption in a building, starting from the most general, i.e., one power meter (PM) per building, to the most specific, i.e., one meter per device. Clearly, there is a tradeoff between precision of measuring and costs of installed and maintained devices.

We observe device-level PMs to know precisely all the state of devices being surveyed. We consider this scenario as a benchmark (ground truth) due to its intrusiveness and costs (i.e., requiring single PM per-appliance). We also observe aggregated power consumption at the room-level, which is slightly less informative. We need to extract the information using a recognition module. The recognition module is responsible for detecting potential switching states and predicting the label of the switching device.

Having compared several predictors and techniques, including NN, for device label prediction [14], we resort to neural network (NN) due to its ability to detect the interactions between predictor variables, the ability to detect nonlinear relationships among variables, and the easy extensibility of the network structure. Neural network is a nonlinear statistical model for regression or classification, typically represented by a network diagram [15]. It works by deriving hidden features *Z* from the input *X* and modeling the target classification *Y* as a function of linear combination of the *Z*.

In this work, we employ NN with three inputs. That is, of each estimated state change, we extract power consumption when a device is in a stable state. We also consider Mean of Absolute Difference (MAD) and Variance, to capture ripples during a device’s active period and to measure how far values are spread out from the steady level, respectively (see [14] for more details). The NN is designed bearing in mind limited training data. In daily life, when a new appliance or device is introduced to a system, a worker should not be burdened with the collection of electricity fingerprints and labels for the new device. Instead, the chosen technique should be suitable for working with limited training data, and a new model should be sufficiently trained using the available data.

The prediction of state and device type is useful in occupancy inference. Let $W_{i}$ where i={1,2,…,m} be a worker in a particular room and let $D^{W_{i}}=\left\{d_{1}^{W_{i}},\ldots ,d_{n}^{W_{i}}\right\}$ be the set of $W_{i}$’s devices. Then the occupancy state of worker $W_{i}$ is a binary state where:

(1) SWi=present,if(d1Wi:ON∪…∪dnWi:ON)absent,otherwise

### 2.2. Occupancy Inference from Received Signal Strength (RSS)

We utilize the worker’s mobile phone as a sensor to discover BLE package broadcasted by beacon nodes and perform self-localization. Let *X* be the input with *n*-dimensional real number, each dimension represents a beacon and theoretically ranges between 0 dBm (excellent signal) and −∞ dBm (no signal). Let $R=[R_{1},R_{2},\ldots ,R_{k}]$ be a set of room labels. The classification task is to identify room label $R_{pred}$, with $R_{pred}\in R$, given *X* measured by mobile device of each worker.

To relate classified room labels to occupancy, we make use of a workspace map. The map indicates the work space $R_{wspace}$ of each worker. This information is commonly maintained by building administrators for various purposes, e.g., letter and parcel handling. Given this knowledge, we can infer an individual occupancy state relative to his/her work space. Formally, it is stated as follows:

(2) SWi=present,if(Rpred==Rwspace)absent,otherwise

We use the *k*-Nearest Neighbor technique to identify a room label given the signal strength indication of BLE nodes. That is, one of the simplest techniques that works by finding a number of labeled samples nearest to a query and predict the class label with the highest votes [16].

As not all of BLE beacons signal will be discovered by a mobile phone, there will be missing data of undiscovered BLE nodes in *X* (no signal or extremely weak signal −∞ dBm). To deal with this, we use cosine distance as an indication of similarity [17]. We use seven types of features listed in Table 1, where *N* are data points per-window *t* for each *N*-dim beacon.

These features are:

- Mean, the average RSS in a moving time window;
- Mode, the most frequently appearing RSS in a time window;
- Standard deviation, the dispersion or variation of a set of RSS values from its mean in a time window;
- Maximum, the strongest RSS value in a time window;
- Difference, the signal strength difference between the average RSS of the current time window and any previous time window;
- isDiscovered, the binary information indicating whether particular beacons are discovered or not; and
- isStrongest, the binary information indicating beacon node with the highest RSS.

It is worth noting that no calibration is needed. The only thing we need is to provide examples to train the models (i.e., one for recognizing the active appliances and another one for recognizing room-level position from RSS). The model for room positioning is built at setup using only a single mobile phone, and not by all users with their phone to avoid burdening the users. Once the models are built and performing reasonably well (see Section 3.3 and Section 3.4), we can perform occupancy detection without further calibration.

### 2.3. Occupancy Inference from Fusion Using Dempster-Shafer Theory of Evidence

Given two completely independent sensors measuring the same phenomenon, we can combine the results as a final output to achieve more accurate environment interpretation, making use of the advantages of each sensory modality. To achieve such result, we opt to use Dempster-Shafer information theory.

#### 2.3.1. Dempster-Shafer Information Theory

Dempster-Shafer Theory of Evidence (DST) is an evidential reasoning approach for dealing with uncertainty or imprecision in a hypothesis [18]. In the scope of sensor fusion, the observation of each sensor can be evidence to characterize the possible states of the system (the hypothesis). In DST reasoning, the basic hypotheses are called frame of discernment θ, that is, all hypothesis elements that are not further dividable. All possible combinations of the elements θ are the power set Θ. For example, if we define set θ={present,absent}, then the hypothesis set space consists of four possibilities, Θ={∅,{absent},{present},{absent,present}}. The hypothesis of {absent,present} can be thought as an unable-to-infer condition between states. For certain models this is possible.

Based on the observed ’evidence’(*E*) from each sensor, the system can assign ’believe’ over the possible hypothesis Θ. Similar to probability, the total belief is 1. The assignment of belief from sensor-*i* is called the Probability Mass Assignment $m_{i}$. The belief of any hypothesis *H* is defined as the sum of all evidence $E_{k}$ that supports hypothesis *H* and the sub-hypotheses nested in *H* [19], as given in Equation (3).

(3) $$Belief_{i}\left(H\right)=\sum_{E_{k}\subseteq H}m_{i}\left(E_{k}\right)$$

Given observation evidence from multiple sensors, the Dempster-Shafer combination rule provides a mechanism to fuse probability masses of the observation of sensor-*i* ($m_{i}$) and sensor-*j* ($m_{j}$) as follows:

(4) $$Belief\left(A\right)=m_{i}⊕m_{j}\left(A\right)=\frac{\sum_{A_{k}\cap A_{k^{\prime }}=A}m_{i}\left(A_{k}\right)\cdot m_{j}\left(A_{k^{\prime }}\right)}{1-K},$$

$$\mathrm{where}\;K=\sum_{A_{l}\cap A_{l^{\prime }}=\emptyset }m_{i}\left(A_{l}\right)\cdot m_{j}\left(A_{l^{\prime }}\right)$$

Based on the believe of sensor-*i* and sensor-*j* generating proposition *A*, we can compute the combined believe of proposition *A* using the combination rule of Equation (4). This value is normalized by 1−K, where *K* indicates conflicts among the sources to be combined (or in other words, all combined evidence that does not match to the proposition *A*). For example, the $Belief(S^{W1}:present)$ is computed from the products of belief that the sensory modalities identify the W1 presence. The conflict factor *K* represents the disagreement of the two sensors towards proposition that W1 is present, such as $m_{i}\left(present\right)\times m_{j}\left(absent\right)$ and $m_{i}\left(absent\right)\times m_{j}\left(present\right)$.

In the case where all hypotheses are singletons and mutually exclusive, the combination rule will produce identical results with Bayesian theory. Given evidence *E* observed by both sensor-*i* and sensor-*j*, the probability of hypothesis *H* is:.

(5) $$P\left(H_{i}|E\right)=\frac{P\left(E|H_{i}\right)\cdot P\left(H_{i}\right)}{\sum_{i}^{j}P\left(E|H_{i}\right)\cdot P\left(H_{i}\right)}$$

where $P(H_{i})$ is the a priori probability from sensor-*i* that the evidence to support hypothesis *H* was observed and $P(E|H_{i})$ is the likelihood that the same evidence was also observed by sensor-*j*. For example, in the case of occupancy, the only possible state must be present or absent (any one of them but not both). Then the probability of being present ($H_{i}$) which is detected by sensor-1 becomes a priori probability, while the probability of the same evidence observed by sensor-2 forming the same hypothesis (i.e., being present) would be the likelihood that it is happening.

#### 2.3.2. Probability Mass Assignment

To fuse independent sensors using the combination rule, we need to build the Probability Mass Assignment as our belief based on pieces of evidence given by a sensor. With respect to room-level PM, the PMA is computed based on our trust in such sensors. The trust can be derived from experimental data about identifying the sensor ability to recognize presence/absence of a particular worker. Such a heuristic way is quite common in the determination of probability, see for example [20]. Concretely, we assign masses based on how closely the identified devices agree with the real occupancy of the owner in the past. The way how we identify the closeness is similar to [21]. We count the frequency of agreement or true positive, false positive (FP), null agreement (NA), and false negative (FN) between the real occupancy and the prediction of device activation. The real occupancy is defined as high precision occupancy state that can be obtained either from manual user input or per-device power meter. The prediction of activated device is derived from the device recognition module of room-level power meter.

**Figure 1:**
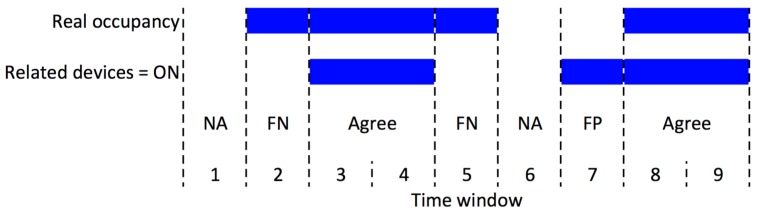
The illustration of agreement between two series: real occupancy of a worker and the prediction of activated devices belongs to the corresponding worker, adapted from [21].

As shown in Figure 1, the agreement/null agreement is the condition when the prediction of active/not active devices agrees with the actual occupancy (presence/absence) of the corresponding worker. FP is counted when the prediction of active device is incorrect, in other words, some devices are predicted as active when the owner does not occupy the workspace. When a particular worker is present, but no one related devices is active, we count this as an FN.

The belief of $W_{i}$’s presence given corresponding devices $d_{j}^{W_{i}}$ are active is computed based on true positive occupancy, normalized to all positive occupancy predictions, while the belief of absence given the corresponding devices $d_{j}^{W_{i}}$ are inactive is computed based on true negative occupancy normalized to all negative occupancy predictions.

Based on the aforementioned approach, we consider the past data (19th April to 1st May, 2017) to assign the probability masses of room-level power meter specific to the particular users. The higher agreement between users’ Presence/Absence and devices’ ON/OFF, the higher probability masses assigned to the corresponding users, as shown in Table 2.

Concerning the BLE-beacon, we calculate the PMA using the conditional probability formula relative to the nearest neighbor instances, as shown in Equation (6). It is based on the probability that indicates the likelihood that a label comes from a particular class. Given the RSS $X_{new}$, the probability of a worker being in room $R_{pred}$ can be computed as the sum of the weight of *i*-nearest neighbor, where its label is equal to room $R_{pred}$ divided by the sum of weights of *i*-nearest neighbors:

(6) $$p\left(R_{pred}|X_{new}\right)=\frac{\sum_{i\in knn}\omega \left(i\right)\cdot 1_{Y(X\left(i\right)=R_{pred})}}{\sum_{i\in knn}\omega \left(i\right)}$$

where knn is *k*-nearest neighbors to the observed $X_{new}$, $R_{pred}$ be a predicted room label, and ω are the weights of the points in the training predictor *X*. When the probability $p(R_{pred}|X_{new})\geq$ confidence level λ, our belief in this sensory modality is very high. Thus, we bypass the sensor fusion and consider BLE-beacon as the final result. Based on the empirical observation, we confidence when 4 of 5 nearest neighbors have the same class label referring to $R_{pred}$. Hence, in *k*-NN with *k* = 5, we choose λ=0.79.

**Figure A1:**
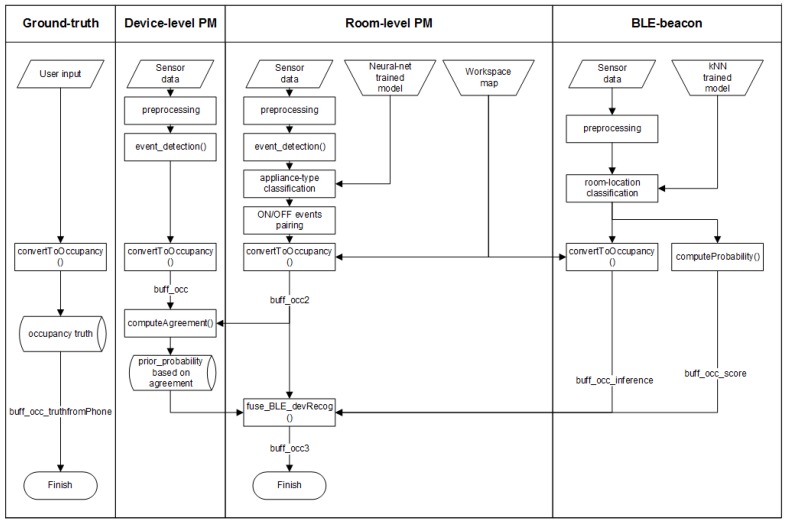
System design from the sensor type perspective.

The overall system design from the perspective of sensor type is shown in Figure A1. The parallelogram symbolizes data source, trapezoid symbolizes supplementary knowledge (e.g., trained models and workspace mapping), and rectangular- and cylindrical-shaped are associated with data processing and data storage, respectively. The occupancy ground truth is collected using an application running on a mobile phone. The only process applied to the ground truth is converting input room-level position to binary occupancy, that is, whether or not a worker is present in his workspace. This output is useful as a comparison to the predicted occupancy. To obtain occupancy from the PMs, firstly we need to detect switching states from the preprocessed data. As for device-level PM, the accurate occupancy state *buff_occ* can be obtained immediately from each power meter attached on every appliance. This process is done using conversion as shown in Equation (1). As for room-level PM, we need to classify the appliance type and to find ON/OFF switching pairs. This step is followed by occupancy conversion as in Equation (1) to obtain occupancy state *buff_occ2*. Based on the agreement between *buff_occ* and *buff_occ2*, we compute prior belief of room-level PM modality and save the results for further process. Room classification using received beacon signal strength is processed through several pre-processes, i.e., feature extraction through a window and decibel-to-magnitude conversion. The data are passed to a classifier whose the model is pre-trained using only a single phone. The classification output is then used to infer occupancy *buff_occ_inference* and to compute the probability as in Equation (6). The final process includes the fusion process from room-level PM and BLE-beacon to achieve fused result *buff_occ3*.

## 3. Experiment

We design an experiment in a living lab setup in our own offices on the fifth floor of the Bernoulli building on the Zernike Campus of the University of Groningen, The Netherlands, to study how well room-level power meters and mobile phones can be used for occupancy detection.

### 3.1. Setup

**Figure 2:**
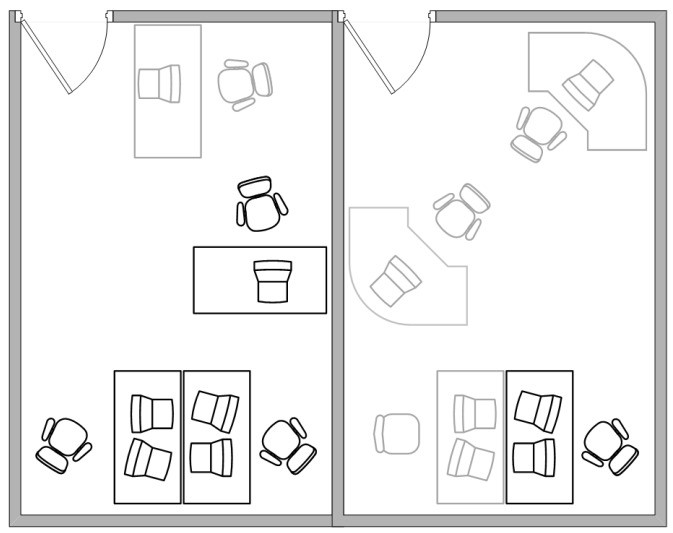
The layout of shared workspaces, Room-2 and Room-3.

We consider shared workspaces with 4 workers, as shown in Figure 2. The gray work desks indicate empty or rarely occupied workspaces that are excluded from the experiment.

All workers have a mobile phone installed with an application to measure the signal strength from Estimote BLE beacons [22] and to report the truth room-level position by pushing a button when he/she moves to the other room. There are 12 beacons deployed on the ceiling of four rooms and of the hallway. These beacon nodes are configured to transmit low power signals (i.e., −20 dBm) with 950 ms broadcasting interval. The used sensors are listed in Table 3.

We deploy plug power meters from Plugwise [23] both on an incoming electrical line (room-level) and on each device (device-level) associated with an individual. We record the room-level PM and the aggregation of per-device power consumptions and find no noticeable difference between the two. Hence, we use the superposition signal as an aggregated power load to be analyzed. The per-device PM measurement is also useful to generate the ground truths of device activation.

The interval between two consecutive power measurements is 10 s. Such an interval is set to assure the data pooler has enough time to receive data from all plugs. If less than 12 values are missing, e.g., due to network failure, we replace the missing values with the latest available data point. Otherwise, we change the missing values to 1.023 Watt, to represent the load of a single Plugwise node (i.e., due to hardware noise, an idle Plugwise measures ranges from zero to about 2 Watt).

**Figure 3:**
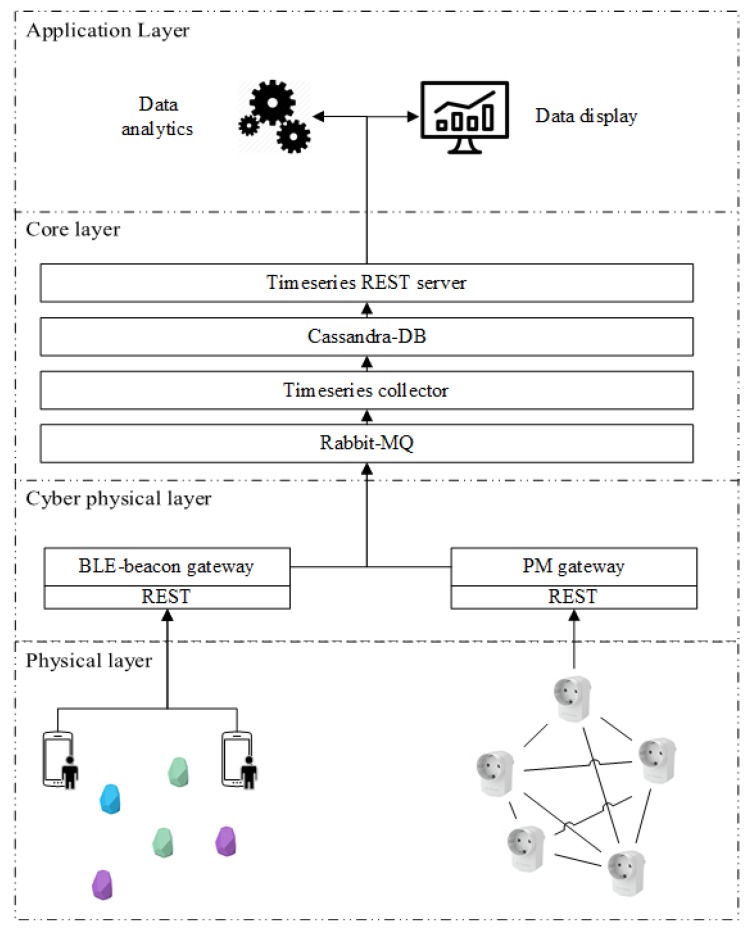
System architecture.

The raw data coming from the sensors flow to a dedicated server, shown in Figure 3. From the physical layer, where various sensors are deployed in the environment, the raw data are sent to the corresponding gateway via a REST interface. The gateways are responsible for bridging the very specific protocol used by sensory modalities (e.g., wireless Zigbee mesh network utilized by Plugwise) to communicate with the upper layer. When receiving new raw data, the gateway publishes them to RabbitMQ [24], a highly reliable and interoperable messaging system based on AMQP standard [25]. We develop a time-series data collector service to collect the data from Rabbit-MQ and store data immediately to an Apache Cassandra database [26].We also provide a time-series REST server to read data from the database by calling the REST service. Once the infrastructure is set up, we are ready to collect and process the data to extract higher-level context; occupancy in our case.

**Figure 4:**
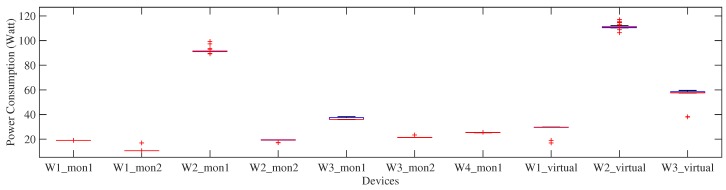
The power consumption of 10 device labels extracted from transition events. The red lines represent median value, blue box plots represent data distribution, and red plus signs mark outliers.

We consider office devices that directly correlate to occupancy. We choose various types of monitor screens being in the workspace to be recognized. There are seven physical monitors belonging to all test subjects. We also define virtualdevice as the set of multiple physical devices belonging to the same subject end that are activated simultaneously, or, in other words, it is the sum of the power of each of its composing appliances. In total there are ten devices (i.e., seven physical- and three virtual-devices) introduced to the classifier in training phase, as shown in Figure 4.

While particular monitor has a noticeable power consumption (e.g., Worker W2’s monitor-1), some other devices (e.g., monitor-2 belonging to Worker W2 have similar power consumption to monitor-2 belonging to worker W3. Some outliers on the average power consumption of the respective devices are also observed, indicated as the red plus sign markers. W1_virtual,W2_virtual,W3_virtual represent virtual device classes belonging to W1,W2,W3, respectively.

### 3.2. Metrics

To evaluate the proposed system, we measure how good the approach is in occupancy detection. To avoid trivial results, we only consider inference performance during work-hours, instead of 24 h. We define the work-hours based on common observation, that is, between 7 a.m. and 9 p.m. The default state of a test subject is absent unless there is an evidence to infer the subject’s presence.

Unless explicitly mentioned, we compute the metric based on 210 time-windows during 14 work hours per day. The time-window is calculated as: $\frac{h\_work\_hours*60\;min}{window\;period(in\;minutes)}$.

The room-label classification from RSS is performed through five minutes moving window. It comprises data points in the four minutes window and one minute overlap with the previous window. We consider such a window size as the occupancy in office spaces is not frequently changing, reducing the computational demands of wider window sizes. As for device recognition, we classify device state based on detected events. We classify a device activation state based on two events that are labeled as consecutive ON and OFF states with the same device ID. For example, at time t=3 an event is classified as device-A with state ON and at the time t=5 the event is also classified as device-A with state OFF, then we set state device-A at $t_{a}=3,4,5$ as active. We thus align the result to the room-label classification in the same range moving window for the fusion process. To quantify the performance of our approach, we consider several metrics, such as accuracy, precision, recall, and F-measure.

#### 3.2.1. Accuracy

Accuracy is defined as the number of windows that are correctly predicted during the day divided by the total number of predictions made. Concretely, it is defined as:

(7) $$Accuracy=\frac{TP+TN}{TP+TN+FP+FN}$$

where True Positive (TP)/True Negative (TN) is the number of windows that present/absent states are correctly detected, and False Positive (FP)/False Negative (FN) is the number of windows that present/absent states are miss-classified.

It is worth noting that we treat inconclusive results as errors. For example, if a window is classified as an occupancy in Room $R_{4}$ while the available ground truth has a different value (i.e., being elsewhere other than Room $R_{4}$), then it is an inconclusive result and we count this as an error.

#### 3.2.2. Precision and Recall

As accuracy itself is insufficient to provide information about classifier’s performance (e.g., high classification accuracy can be misleading as the model returns the majority class for all predictions, but actually has a low predictive power), we also compute Precision (also referred to as Positive Predictive Value) and Recall (also referred to as Sensitivity or True Positive Rate). They are defined as follows:

- Precision is the rate of True Positive over all events detected by the system, regardless the truth, formally:
  (8) $$Precision=\frac{TP}{TP+FP}$$
- Recall is defined as the proportion of real events that are correctly identified, formally:
  (9) $$Sensitivity=\frac{TP}{real\;positive}=\frac{TP}{TP+FN}$$

#### 3.2.3. F-Measure

The F-measure is the weighted harmonic mean of Precision and Recall:

(10) $$F-measure=\frac{\left(1+\beta^{2}\right)*recall*precision}{\beta^{2}*recall+precision}$$

where β=1, that is, assigning an equal weight to recall and precision.

### 3.3. On the Comparison of an Existing Technique

To review the performance of existing occupancy inference through BLE before doing fusion, we reproduce the work of Filippoupolitis et al. [12] on our dataset. It is chosen due to its similarity to ours in terms of experimental setup and proposed technique (i.e., *k*-NN). Our work differs in similarity distance, classification features (see Section 2.2), and data imbalance. Furthermore, we explore the occupancy inference from BLE data obtained from other mobile phones utilizing a model trained from only one mobile phone (i.e., the phone belongs to W1 using data from the 9th of March to the 2nd of May 2017), see Section 4.1.

For providing a fair comparison, we select a dataset from one mobile phone and apply random data down-sampling to our highly imbalanced dataset. The number of down-sampled data points per class is based on the smallest number of available data points of a class, resulting in the number of data points: 969, 504, and 280 for the window size of 5, 10, and 20 samples, respectively. We thus use 10-fold cross validation to test the performance and repeat ten different random samples for each window size. The comparison of [12] and our approach is shown in Table 4. Our proposed *k*-NN method using cosine distance has an accuracy which is about 7% higher than when using the same method based on standard Euclidean distance (as done in [12]) for all window sizes taken into account. The F-measure metric also shows the same trend. In fact, the cosine distance-based *k*-NN performs slightly higher than the one with Euclidean distance.

### 3.4. On the Performance of Device Recognition

To review the pattern existence on the electricity fingerprints, we firstly detect events that are triggered when potential switching states occur. For this purpose, we analyze device-level plug meter data to preserve feature clarity. We extract 1208 switching state instances (see Section 2.1) from the data we have (147 days, 13 March–30 October 2017). Each instance has power-level, MAD, and variance as features. We mine the pattern of device switching states through this data using 10-fold cross validation with the neural-network algorithm [15]. The network comprises a single hidden layer with 20 neurons. We repeat the experiment ten times and summarize the results in Table 5. The table shows that we can capture the patterns of switching events of our devices. Based on this result, we verify that the features taken into account can reveal the patterns of device switching events.

In the rest of this paper, we only use a small portion of this data (i.e., 317 instances, 13–31 March 2017) in supervising a model, to reflect limited available training data in daily usage. We thus use the generated model to recognize devices from unseen data of aggregated power loads to infer occupancy.

## 4. Results

Based on the approach introduced in Section 2, we perform occupancy inference using device- and room-level plug meter, BLE beacon, and fusion between room-level plug meter and BLE beacon.

### 4.1. Occupancy Inference

With device-level power meterings, we can infer individual occupancy with accuracy of 90–98%, see ’Actual’ in Table 6. As this approach is considerably intrusive (i.e., requires single power meter per-device), we only consider this modality as a benchmark to show the best possible occupancy inference using per-device electricity consumption.

The occupancy inference experiment for worker W1 covers 43 work days. Having only the aggregated power consumption, we can infer with 67.90% accuracy the presence of W1. The precision and recall are 66.96% and 96.71%, respectively, resulting in 77.40% F-measure. Given BLE information, the system can infer occupancy with 88.74% accuracy. The F-measure improves, reaching 90.88%. This is the highest performance that was achieved by low intrusive sensory inference. Compared to the inference of device-level power meter, the result drops by 3%. Even though the proposed fusion cannot outperform the overall occupancy of W1 using BLE-based inference, the gap is only about 1%, reaching 87.12%.

The occupancy inference of W2 consists of 27 work days. The highest performance is achieved by using the fusion process of the room-level power meter and BLE, reaching 90% accuracy and 91.49% F-measure. This is comparable to the result of occupancy inference by per-device power metering. The fusion process considerably improves the BLE-based inference of W2 by 11%, and slightly better than occupancy inference using room-level power meter (89% accuracy).

We observe the presence of W3 over 6 days. The performance of occupancy inferred using room-level power metering is comparable to the performance of inference using BLE, about 79% accuracy and 86% F-measure. The implementation of fusion slightly improves the overall performance, reaching 81.75% accuracy and 87.40% F-measure. The recall of the W3 occupancy inference is close to 100% for all modalities and becomes the highest recall in this work.

The experiment of occupancy inference of worker W4 covers 10 work days. We obtain 79.24% accuracy and 86.92% F-measure, respectively, by having only the device-level power consumption. By using BLE, the occupancy of the same person can be better predicted, reaching 89.47% accuracy and 92.86% F-measure. Compared to the inference of device-level power meter, the result is not much different in terms of both accuracy and F-measure. That is, about 3% less than the accuracy and F-measure of appliance-level inference.

### 4.2. More Detail in Sensor Fusion

To have better insight in sensor fusion, we present and discuss inference results of all subjects in the experiment. To this end, we pick the best and worst cases.

#### 4.2.1. Time Sequence Occupancy Inference

**Figure 5:**
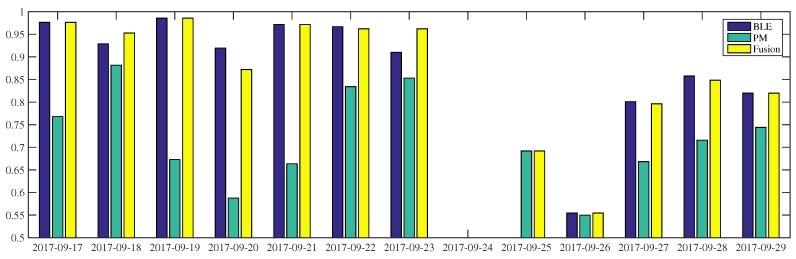
The occupancy inference of *W1* using BLE beacons, room-level electricity measurement, and fusion during 2 weeks surveillance.

**Figure 6:**
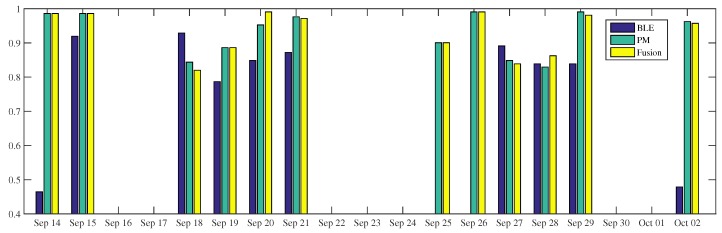
The occupancy inference of *W2* using BLE beacons, room-level electricity measurement, and fusion during 2 weeks surveillance.

**Figure 7:**
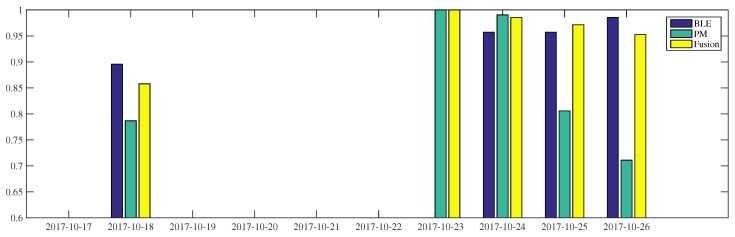
The occupancy inference of *W4* using BLE beacons, room-level electricity measurement, and fusion during 1 week surveillance.

Figure 5, Figure 6 and Figure 7 show the occupancy inference of workers over several days. As shown in Figure 5, all occupancy inferences of W1 using BLE outperform power-meter-based inference. This result is confirmed by Table 6: the BLE approach is better than the other low intrusive sensors. In the first half portion, the BLE-based inference can reach more than 90% accuracy while in the last three days (i.e., 27–29 September) the accuracy of the same modality drops by 5–10%. With respect to power-meter-based inference, we can see fluctuations in the two weeks observation. The proposed fusion makes use of the BLE modality to perform better along the period. On some days (i.e., 18 and 23 September), the fusion inferences are even higher than their composed modalities. On 23 September, the BLE and power-meter-based inference results 91% and 85%, respectively, the fusion inference of these sensors reaches 96% accuracy. When the BLE modality is unavailable, e.g., due to system failures or a deactivated module, the system can still infer occupancy, as it occurred on the 25th of September. However, when all modalities fail to infer occupancy, the proposed system is unable to improve the performance, as it occurred on the 26th of September. Furthermore, the failure of power-meter in inferring occupancy can worsen the fusion inference which falls by 5% accuracy on 20 September, reaching 87%.

In general, the occupancy inference of W2 using power-meters is better than using BLEs, (Figure 6). Even though the BLE-based inference is leading in the particular time (e.g., on the 18th and 27th September), such domination does not happen very often. This is also confirmed by the overall occupancy inference, Table 6. The proposed fusion makes use of the power-meter modality to perform better during the period. When the BLE-based inference does not work (e.g., on the 14th of September and 2nd of October) or is not available (25–26 September), the fusion results are still above 90%. The fusion inference is higher than BLE and power-meter, that is, reaching 99% and 86% accuracy on the 20th and 28th of September, respectively. On the 18th of September, the fusion of the two modalities does not bring any benefit. In fact, one has worse inference performance than any single sensor, reaching 82% accuracy.

As shown in Figure 7, for W4 the fusion process achieves occupancy inference more than 95% accuracy, during the 23–26th of October, and about 85% on the 18th of October. The BLE-based occupancy inference of this worker delivers at least 90% accuracy during the period. The power-meter-based inference presents considerable fluctuations, in the range of 70–100% accuracy. When the power-meter result is at its lowest performance, as on 26 October, the fusion process improves the occupancy inference reaching 90%. On the 25th of October, the BLE and power-meter-based inferences show 95% and 80% accuracy, respectively. By fusing this result, the final inference accuracy reaches 97%.

#### 4.2.2. Inference Details

Next, we draw the detailed occupancy inference of power meter, BLE, and sensor fusion.

**Figure 8:**
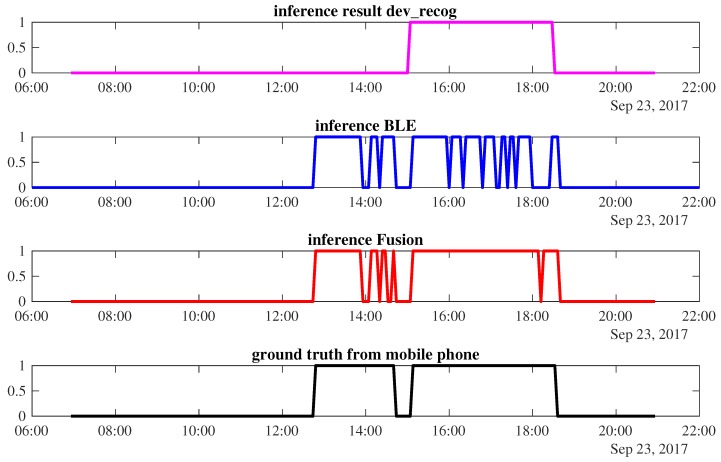
The occupancy inference of *W1* using different types of sensors and its ground truth on the 23 September 2017.

*Case 1*: Sensor fusion improves the final result. Figure 8 shows the occupancy prediction of W1 on the 23rd of September. The power-meter-based inference infers occupancy only from 15:00 to 18:32, while occupancy during 12:48 to 14:40 is misclassified by this modality. The BLE correctly predicts when the worker arrives and leaves. However, in the middle of the occupancy period, it frequently detects false absence, resulting in fluctuations in the occupancy prediction. Sensor fusion can improve the accuracy of the final results by correcting the power meter inference and the fluctuation of the BLE prediction. An accuracy of 96.2% is achievable by such a fusion combination.

**Figure 9:**
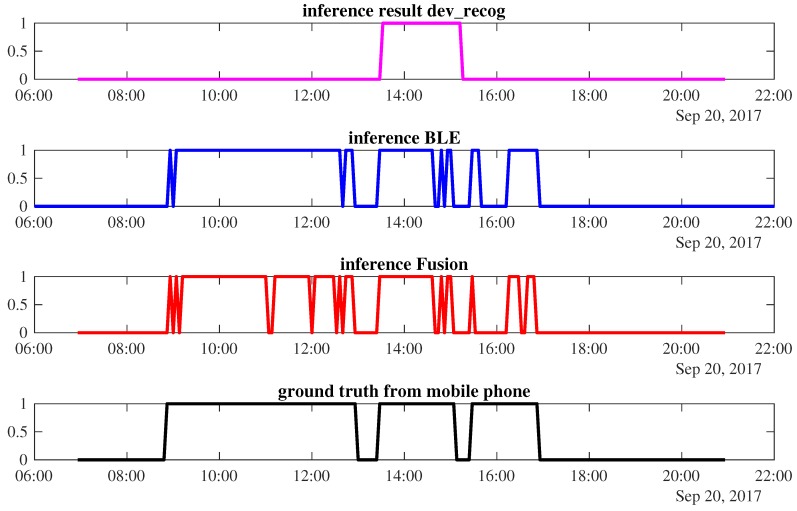
The occupancy inference of *W1* using different types of sensors and its ground truth on the 20 September 2017.

*Case 2*: Sensor fusion is unable to improve the final result. As shown in Figure 9, the power-meter-based occupancy inference detects only W1’s presence from 13:32 to 15:12, resulting in 58.77% accuracy. The BLE inference is better as it provides 91.94% accuracy. However, when we fuse both sensors, the final occupancy result does not improve. During the occupancy period of 09:04–12:36, there are several time windows that predict the corresponding worker leaves. It happens between 11:00–11:12 and 11:56–12:00; 16:28–16:40 as shown in red. This lowers the final accuracy performance to about 87.2%.

## 5. Discussion

From the results, we can see that neither BLE-beacon nor room-level PM is the best predictor for every single worker. W1 and W4 occupancy is better predicted using BLE inference, while W2 is better predicted using room-level PM. As for W3, both sensors basically have the same accuracy. We conclude that the high performance of W2 occupancy is because our approach takes benefit from high-power consumption of W2’s device (see Figure 3) that is easily separable from the room-level power meter (this fact delivers higher precision than recall for W2’s occupancy). The power consumption of others’ devices is more difficult to distinguish due to similar consumed power and leads to worse occupancy inference than BLE inference. A justification of good BLE-based occupancy inference (e.g., in W1 and W4) is the appropriateness of the classification model. The classification model is built on training occupancy data recorded from W1’s mobile phone, hence, it produces a reasonably good performance of 43 work days. The same classification model has also performed well on the ten work days of W4, giving 89% accuracy and 93% F-measure. However, such a model does not deliver a very satisfying result in the occupancy of W2 and W3. We believe this is due to RSSI variation among mobile phones [10]. Furthermore, some factors that could affect the BLE-based inference are signal interference on the 2.4 GHz radio wave and the orientation of mobile phones affecting the line-of-sight condition between BLE beacons and phone receiver. The details of these disturbances are out of scope of the present work.

We can also observe that the occupancy inference using all sensory information provides lower precision than recall, except for the occupancy of W2. This result indicates that the approach is better in negative occupancy (absence) than positive occupancy inference (presence). The reason is that absence is easier to recognize, e.g., absence is a default state when BLE signal strength is not discovered or when start and end device activation pair are not found or matched (see Section 2.1). Interestingly, we can observe that the recall of W2 device-level based inference is the only one with a value below 90%, while the recall of W3 occupancy reaches 99%. This means that W2 inference has a high number of false negatives while W3 has almost no false negatives. This indicates that in W3 occupancy inference, the system can reliably predict when W3 leaves. For example, she/he always makes the computer standby/turned off when she/he goes away from the office.

Our fusion approach can mostly select better evidence among available sensory modalities (i.e., BLE-beacons or device-recognition), even though the final averaged result is not always better than its composed sensor. A reason why the fusion sensor does not bring overall better results for all worker is that the fusion process highly depends on the belief of its individual sensory inference. For example, a correct BLE-based occupancy inference can present incorrect final fused-occupancy if the evidence of BLE is not strong enough in supporting its output. The concrete example is *k*-NN with k=5, where three nearest neighbors vote for positive occupancy of a corresponding room and the other nearest neighbors are the representation of the other rooms. In this case, the fusion decision might be the negative occupancy if another sensor is more confident with negative occupancy. Thus, the fused occupancy result presents lower performance than BLE. The occurrence of precise prediction can also affect the fusion result being worse than the inference of individual sensor. That is, the timing (or period) when a correct prediction occurred during a day. The fusion will perform better than each sensor if a single sensory modality contributes positively in different time frames. In case 1 (see Section 4.2.2), the room-level power meter better predicts in the afternoon but fails to infer occupancy in the morning. The BLE-based inference estimates better in the time when the occupancy detection based on room-level PM does not give a correct prediction, thus providing better final inference.

In the current work, the prior belief of BLE beacon is based on the nearest neighbor instances, for the room-level PM the prior belief is based on the agreement of previous experimental data that is kept in storage (see Figure A1). The room-level PM belief assignment might not be the best approach for determining probability to make a decision. The value will depend on the previous samples that can fluctuate depending on the chosen data. Another limitation that we plan to address in the future is the direct portability of the system and ground truth collection procedure. The performance recorded for the experiments reported here might vary when having office layouts and a number of occupants which vary. Moreover, as the ground truths are collected on the basis of proactive users’ feedback, unmotivated or distracted users may make mistakes in reporting their state. In our case, we have relied on very dedicated individuals, but there is no guarantee that the ground truth has no deviations from the actual situation. Despite these limitations, the application of power meter as occupancy can be a feasible solution on the small to medium scale, e.g., room- or zone-level, as compensation of low-power appliances to be recognized. The scalability of this approach can be stretched out by a per-room PM installation.

## 6. Related Work

Related work to this research vary from the location-based electricity load disaggregation to the occupancy inference system based on energy consumption and BLE. These are summarized in Table 7.

Kleiminger et al. researched the use of power meters as an indication of human presence [11]. They study binary occupancy (i.e., home or away) of houses based on aggregated power consumption of each residence. The average performance is 86% and 83% accuracy using *k*-NN and HMM, respectively. This is measured from 6 am to 10 pm. However, their approach only focuses on coarse occupancy without further expansion to individual presence.

LocED, Location-aware Energy Disaggregation, is a framework of energy disaggregation based on known occupant location [27]. The main goal of the project is to disaggregate power consumption by making use of location estimation of an occupant. The location information is derived from BLE and WiFi AP. The experiment takes place in a residential area with 6 rooms. There are 13 deployed beacons around the house, i.e., Room1 (2BLE), Room2 (2BLE), LivingRoom (3BLE), Kitchen (3BLE), Store Room (2BLE), and Outside (1BLE). The Bayesian-based approach is used to infer room-level location. As their research does not focus on the occupancy detection, they assume that the inferred location is trustful. Unfortunately, they did not provide the location inference performance in their report. While exploiting the same sensor types (i.e., power meter and BLE), our work goes in the opposite direction, that is, we utilize electricity consumption measurement to infer occupancy.

Blue sentinel, a BLE-based room occupancy detection system, has been developed by Conte et al. [13]. They propose a modification of the iBeacon protocol on the Apple iOS operating system, that by default is unable to continuously track the users. The modification is that of forcing the OS to wake up the application more frequently than standard by advertising beacons in a cyclic sequence. They employ 3 beacon nodes to classify 3 room labels with *k*-NN and decision trees. They collect 1234 instances and validate them using 10-cross validation resulting in the accuracy of 83.4% without giving details at the individual room occupancy level.

Individual room occupancy was proposed in Filippoupolitis et al. using three different approaches, namely Logistic Regression, *k*-NN, and SVM with various window sizes [12]. In total, the authors use 8 beacons to detect occupancy of 10 areas that are divided into two independent sectors. The reported accuracy is between 80% and 100%, depending on the room. This result is obtained from 10-cross validation of 1700 data points (350 instances per-class). Both Conte et al. and Filippoupolitis et al. consider only one mobile phone to measure the signal strength and use the same phone to test through cross-validation method. From the various studies it emerges that the RSS appears to be measured inconsistently across mobile devices [10]. In our experiments, we use four distinct types of phones.

Girolami et al. propose a supervised occupancy detection by exploiting two different BLE transmitted signal strengths (i.e., −18 dBm and 3 dBm) [28]. Each tracked user needs to bring a BLE transmitter with him/her and BLE receivers are deployed in each room. This makes sure that the BLE equipments are homogeneous (one same type) as provided by building managers, but require people to bring an extra device. On the contrary, in our approach, people only need to carry their mobile phone, but as a trade off, there are heterogeneous BLE receivers. They utilize SVM and Random forest classification techniques. They set a controlled scenario using markers and expect users to record their movements to collect the ground truth. The accuracy is of about 72–84% for 3 rooms plus a corridor for various window size for about 1600 classified instances. It is unclear however, the portion between train and test data.

Solving the occupancy detection problem by utilizing several sensory modalities and fusing the collected data is not new. Barsocchi et al. recently exploit motion-, noise-, and power consumption-sensors to detect occupancy of two single-occupancy offices [29]. The power metering is exploited by searching for high-value and high-variability power consumptions, represented as a mean and standard deviation of PM readings, as a presence-state representation. To combine the sensory modalities, the authors implement an algorithm inspired by the stigmergy of ant’s pheromone release. The algorithm requires the optimization for two parameters of each sensor, i.e., amplitude intensity and dispersion decay. Finally, they use an equation based on natural exponential function to compute a sensor-specific value that need be summed up to see whether or not it exceeds a pre-determined threshold. An occupancy status is decided when the value is greater than the threshold.

Apart from stigmergy fusion, Dempster-Shafer theory is a popular technique to fuse different sensory modalities. For occupancy sensing, Nesa et al. have experimented with several combination of sensors, such as humidity-, light-, CO${}_{2}$-, and temperature-sensors [30]. They use data from an open dataset [31]. The goal of their work is to infer occupancy of a single room that is occupied by two persons. Several techniques are chosen such as decision tree, gradient boosting, linear discriminant analysis, and sensor fusion using Dempster-Shafer Theory. They propose a formula to compute PMA (or belief) under the assumption that the sensory information follows a normal distribution. From a 6 days training set, they validate their proposed approach in 2 testing sets, each contains 2 and 7 days. The achieved result is satisfying for all sensory combinations that fused with light sensor, that is, about 97% for classification with decision tree, LDA, and DST. For the single sensor inference, the result is 78% and 84% for CO${}_{2}$ and temperature sensor, respectively. However, their solution does not solve the problem that requires occupant identification. For example, of the occupied state inferred, there is no information how many persons are present and who they are.

A finer grain context recognition has been explored by Milenkovic et al. by counting the number of people, detecting office related activities (i.e., work with or without PC), and simulating energy usage [6]. The authors employ motion sensors and appliance level PMs to observe three workspaces with the different number of people, i.e., 1, 3, and 4 people. The main idea of their approach is to detect worker presence indicated by motion sensors followed by investigating activities by exploring whether or not a monitor is being used. Even though [6] and our approach observe the same object (i.e., monitor screen activation), the approach is different. We recognize monitor activation from an aggregated load, rather than thresholding of the appliance-level PMs, in line with our effort to reduce intrusiveness and cost (i.e., number of PMs) as much as possible. Moreover, their presence detection based on PIR sensor results in an accuracy of 75%, 56.3%, and 63.5% (with overall presence and absence 87%, 88.3%, 72%) for private office, 3-persons, and 4-persons, respectively, showing space for improvement, especially in multi-person offices.

## 7. Conclusions

We proposed a person-level occupancy detection approach based on low-intrusive sensors. Our novel approach exploits plug loads information from room-level PM to understand the occupancy context of a building. We also utilize worker’s mobile phones as a receiver of BLE-beacons signal.

We begin with an initial review of each sensory input using cross-validation approach. We show that our selected features and cosine distance outperforms the existing approaches in BLE-based occupancy inference. We also show that the (shallow) neural network dev-recog is better than *k*-NN and Naive-Bayes approach. Of the aforementioned individual sensory inputs, no one type of sensor is the best in predicting entirely four participating subjects, even though for particular workers, each sensor can infer occupancy with up to 89% accuracy.

We improve the robustness of the occupancy detection by fusing room-level PM and BLE-beacons using probability approach, reaching 87–90% accuracy and 89–93% F-measure. As for worker W3, the achieved accuracy is slightly below the occupancy detection of other workers, reaching 82%. However, this result is just over occupancy inference of its individual composed sensor.

The probabilistic approach used in sensor fusion rises the issue of setting up the probability score of a particular sensor. In this work, the probability is our trust in the room-level PM sensor that is assigned based on the past experimental data, and the likelihood that a label comes from a particular class in the BLE beacon sensory modality. As for heuristic at the room-level PM, the approach is not always optimal in improving the fusion results and requires vast amount of historical data to more closely represent the actual sensors’ ability to reveal occupancy. Another issue is the scalability of the occupancy inference using power meters. While this approach is less intrusive, it requires electricity measurements at the room- or zone-level to scale well. This is because we need to limit the number of devices in one measurement place as a trade-off for the low-power appliances to be recognized. The lower amount of energy consumed by a device, the more difficult it is to recognize, as the switching of states of devices can be masked by energy consumption ripples.

We leave probability assignment optimizations to improve final occupancy inference as future work. Additionally, an adaptive trust provisioning mechanism might also be a solution to represent our trust based on sequential historical data, e.g., assigning the trust based on the inference performance in recent few days. We also plan to experiment with other types of office plug devices.

## Figures and Tables

**Table 1:** The 84-dimensional feature space for room-label classification.

Features	Formula
mean	$\mu =\frac{1}{N}\sum_{i=1}^{N}X_{i}$
mode	$\hat{X}=argmax{\left(X_{i}\right)}_{i=1}^{N}$
std. deviation	$X_{std}=\sqrt{\frac{1}{N}\sum_{i=1}^{N}\left(X_{i}-\mu \right){}^{2}}$
max	$X_{max}=max{\left(X_{i}\right)}_{i=1}^{N}$
diff	$X_{diff}=\mu_{t}-\mu_{t-1}$
isDiscovered	$1_{\left(\exists X_{i}\neq \infty ,i\in N\right)},0\;\mathrm{otherwise}$
isStrongest	$1_{\left(max\left(X_{max}\right)\in R^{n}\right)},0\;\mathrm{otherwise}$

**Table 2:** Probability Mass Assignment of room-level Power Meter, specific for worker W1,W2,W3,W4.

Believe	W1_devices		W2_devices		W3_devices		W4_devices	
	ON	OFF	ON	OFF	ON	OFF	ON	OFF
Presence	71.35	23.71	98.50	38.10	83.20	4.03	68.50	13.63
Absence	28.65	76.29	1.50	61.90	16.80	95.97	31.50	86.37

**Table 3:** Available sensors in the living lab.

Id	Phone Type	Plug Meter(s)	BLE Beacons
Room-1	n/a	n/a	2 nodes
Room-2 and 3	n/a	1 room-level	2 + 2 nodes
SocialCorner	n/a	n/a	3 nodes
Hallway	n/a	n/a	3 nodes
W1	Samsung Galaxy S6 edge+	2 device-level	n/a
W2	Samsung Galaxy S6	2 device-level	n/a
W3	Samsung Galaxy A5 (2016)	2 device-level	n/a
W4	LG Nexus 5x	1 device-level	n/a

**Table 4:** Average accuracy and F-measure for occupancy detection based on BLE beacons.

Method (window_size)	Accuracy Avg. (Std.)	F-Measure Avg. (Std.)
euclidean *k*-NN [12] (5)	0.7990 (0.003)	0.7455 (0.009)
euclidean *k*-NN [12] (10)	0.8210 (0.08)	0.7841 (0.013)
euclidean *k*-NN [12] (20)	0.8093 (0.011)	0.7322 (0.023)
cosine *k*-NN (5)	0.8714 (0.005)	0.8117 (0.008)
cosine *k*-NN (10)	0.8984 (0.005)	0.8307 (0.014)
cosine *k*-NN (20)	0.8718 (0.009)	0.8042 (0.016)

**Table 5:** Average accuracy and F-measure of device recognition.

Method	Accuracy Avg. (Std.)	F-Measure Avg. (Std.)
*k*-NN (k=5)	0.9048 (0.004)	0.8221 (0.008)
NB	0.7777 (0.021)	0.2054 (0.0148)
1-layer neural net	0.9283 (0.0071)	0.8582(0.0156)

**Table 6:** Occupancy inference performance per-individual. The best per-individual inference is marked by bold text

Person	Modality	Accuracy	Precision	Recall	F-Measure
W1	Actual	0.9178	0.9151	0.9623	0.9321
	Predicted	0.6790	0.6696	0.9671	0.7740
	BLE	**0.8874**	0.8630	0.9741	**0.9088**
	Fusion	0.8712	0.8429	0.9745	0.8970
W2	Actual	0.9005	0.9458	0.9107	0.9194
	Predicted	0.8907	0.9483	0.8953	0.9096
	BLE	0.7969	0.7563	0.9707	0.8397
	Fusion	**0.9008**	0.9462	0.8989	**0.9149**
W3	Actual	0.9858	0.9867	0.9891	0.9877
	Predicted	0.7915	0.7952	0.9905	0.8665
	BLE	0.7970	0.7565	0.9935	0.8557
	Fusion	**0.8175**	0.7962	0.9907	**0.8740**
W4	Actual	0.9341	0.9472	0.9765	0.9578
	Predicted	0.7924	0.8107	0.9640	0.8692
	BLE	**0.8947**	0.8737	0.9948	**0.9286**
	Fusion	0.8919	0.8745	0.9955	0.9279

**Table 7:** BLE; PM = power meters; Oth = Others.

Ref.	Sensors			Size	Techniques	Quantitative Performance	Pros	Cons
	BLE	PM	Oth					
[11]	-	✔	-	5 houses	*k*-NN, SVM, thresholding, HMM	86% accuracy (*k*-NN)	off-the-shelf power meter	coarse-occupancy
[27]	✔	✔	-	6 rooms	Bayesian- based	-	WiFi and BLE combination	assuming accurate location
[13]	✔	-	-	3 rooms	*k*-NN; Decision Tree	83.4% 10-fold CV	exploration on the iBeacon protocol	no validation in real-life
[12]	✔	-	-	10 rooms	Logistic Regression, *k*-NN, SVM	80–100% 10-fold CV	giving individual room occupancy	training and testing with one and the same mobile phone
[28]	✔	-	-	3 rooms + corridor	SVM; random forest	72–84%	multi-power transmitters	marker’s guided; must bring BLE badge; not clear train-validation-test dataset portion
[29]	-	✔	✔	2 rooms	Stigmergy approach	95% accuracy 70% precision averaged	fusion approach adopted from other field	single-person occupancy, summarizing power consump may reduce info
[30]	-	-	✔	1 room	Decision tree, LDA, and DST	97% (fusion) 78–86% (single sensor)	fusion, multi-person occupancy	disregarding person identity (binary occupancy)
[6]	-	✔	✔	3 rooms	FSM; Layered HMM	72–88% accuracy of presence inference	people counting, activity detection, and energy consumption simulation	no fusion effort, predefined threshold-based, intrusive device-level PM

## Abbreviations

The following abbreviations are used in this manuscript:

BLE	Bluetooth Low Energy
CV	Cross-validation
dev-recog	Device recognition
DST	Dempster-Shafer Theory
LDA	Linear Discriminant Analysis
NB	Naive Bayes
NN	Neural network
Oth	Other
PIR	Passive Infrared (sensor)
PM	Power Meter
PMA	Probability Mass Assignment
RFID	Radio-Frequency Identification
RSS	Received Signal Strength

## Appendix A

Raw data gathered from different sources can reveal the occupancy of a user. These sources are visualized as four columns representing different data inputs considered in our work, as shown in Figure A1. See Section 2 for details.
